# Impact of single‐cell RNA sequencing on understanding immune regulation

**DOI:** 10.1111/jcmm.17493

**Published:** 2022-07-30

**Authors:** Xueli Hu, Xikun Zhou

**Affiliations:** ^1^ State Key Laboratory of Biotherapy and Cancer Center West China Hospital, Sichuan University Chengdu China; ^2^ Collaborative Innovation Center for Biotherapy West China Hospital Chengdu China

**Keywords:** application, immune regulation, immune‐related diseases, impact, scRNA‐seq

## Abstract

Single‐cell RNA sequencing (scRNA‐seq), one of the most powerful technologies, can describe the transcriptomic heterogeneity of single cells and reveal previously unreported cell types or states in complex tissues. With the rapid development of scRNA‐seq, it has renewed our view of cellular heterogeneity and its significance for deeply understanding cell development and function. There are a large number of studies applying scRNA‐seq to investigate the heterogeneity of immune cells and disease pathogenesis, focusing on differences among every individual cell, which have provided novel inspiration for disease therapy and biological processes. In this review, we describe the development of scRNA‐seq and its application in immune‐related physiological states, regulatory mechanisms and diseases. In addition, we further discuss the opportunities and challenges of scRNA‐seq in immune regulation.

## 1 INTRODUCTION

The earliest single‐cell transcription technique can only detect a handful of genes in a single cell with relatively low throughput.^1^ Historically, scRNA‐seq was applied when limited by the number of biological materials such as cells from early embryonic development.^2^ It was not until 2009 that the single‐cell transcriptome was combined with high‐throughput sequencing, which allowed for improved throughput of gene detection and more detected transcripts than chips.^3^ The impact of scRNA‐seq has grown rapidly since ‘single‐cell sequencing’ was selected as the method of 2013, mainly because of its high throughput of droplet or combinatorial indexing approaches and its wide use of advanced equipment, reagents and analysis tools.^4^ In recent years, scRNA‐seq has developed remarkably in the immune systems with the identification of transcriptionally distinct cell types unknown before, which has helped biologists obtain deeper insights into their research areas.^5^, ^6^, ^7^, ^8^

There are many distinct cell lineages localizing in primary and secondary lymphoid organs in immune systems. They also exist in tissues throughout the body and have dynamic abilities to migrate through the peripheral blood.^9^ Recent advances in scRNA‐seq and sophisticated bioinformatic methods have renewed our eyes on understanding immunology, which may help us reveal novel therapeutic targets from individual cells masked previously by bulk analysis. During the past few years, many studies have focused on immune regulation not only in physiological states but also in diseases, including tumours,^10^, ^11^, ^12^ autoimmune diseases^13^ and infectious diseases.^14^

In this review, we present the development and application of scRNA‐seq in the immune regulation of healthy and disease states and further discuss the strengths and weaknesses of scRNA‐seq.

## 2 OVERVIEW OF scRNA‐seq

Single cells are the fundamental units of organisms and play an essential role in biological processes, such as proliferation and differentiation.^15^ These single cells express different genes even when derived from the same tissues or cell line. Therefore, it is important to better understand biological processes and diseases from the single‐cell perspective, which can provide more accurate and sensitive information for researchers. In previous studies, single‐cell technologies with low throughput, such as single‐cell PCR and real‐time PCR, were applied to detect the expression of a particular gene in individual cells.^3^ However, scRNA‐seq has been applied widely and has provided much information not known before.^16^

The workflow of scRNA‐seq is composed of four key steps: isolation of a single‐cell suspension, single‐cell capture and library preparation, sequencing and preliminary analysis and data visualization and interpretation. The major technical challenges of scRNA‐seq are how to increase cell throughput, and key breakthroughs are the need to achieve automatic isolation of single cells, nondirectional amplification of the entire transcriptome from a single cell and the ability to process multiple cells in parallel. Then, it increased from a single digit to tens of thousands of cells experiencing the initial manual separation,^3^ multiplexing method,^17^ integrated fluid circuit^18^ and droplet microfluidic technology.^19^ Barcode, a nucleotide sequence of more than ten bases, was applied to label single cells in the single‐cell micro reaction system. During the process of reverse transcription, all transcripts of the same cell were labelled with the same barcode to conduct mixed library preparation and distinguish the gene expression level of each cell.^20^ Moreover, to avoid the effect of PCR bias on gene expression quantification and make gene quantification more accurate, UMI (unique molecular identifier) was applied.^19^ For gene sequencing, current scRNA‐seq is used to capture RNA molecules carrying Poly (A) through Poly (DT), mainly detecting mRNA and a small part of lncRNA. The range of the detected gene region is generally approximately 50–100 bp at the 3′ end, and it is sensitive to genes with medium and high expression.

Currently available platforms cover many methods: 10x Chromium Genomics,^21^ Drop‐seq,^19^ inDrop,^22^ Seq‐well,^23^ SmartSeq,^24^ SmartSeq2,^25^ MARS‐Seq^26^ and Microwell‐seq.^27^ 10x Chromium Genomics is a droplet‐based microfluidics system that employs much parallelization to increase throughput but reduce labour and reagent costs. Drop‐seq and inDrop apply similar methods to obtain droplets, while they are different in bead manufacturing, barcode compositions and cDNA amplification. Moreover, Seq‐well and Microwell‐seq use microwell arrays to capture single cells, which benefits high‐throughput analysis and relatively low cost. SmartSeq/SmartSeq2 applies full‐length transcriptome coverage with 5’ UMI RNA counting and allows computational reconstruction of RNA molecules. MARS‐seq is a high‐throughput design of CEL‐seq automatically. It was previously the main large‐scale method for scRNA‐seq in immune profiling.^28^ These methods have their own features in mRNA reverse transcription, cDNA amplification, coverage, capture method, cost, imaging capability and UMI (Table 1). The application of these methods has its own advantages and disadvantages. MARS‐seq uses fluorescence‐activated cell sorting (FACS) of single cells into multiwell plates and subsequent automated processing, which is suitable for identifying novel cell populations. Drop‐seq uses UMI and barcodes to mark mRNA from individual cells, which then facilitates pooled sequencing from multiple cells. Currently, Drop‐seq is more popular as it is a high‐throughput platform for discovering new cell types, constructing cell differentiation trajectories, molecular mapping of differentiation processes, embryonic development and more. However, Smart‐seq2 enables the detection of a higher number of genes, approximately 9000 per cell. It is useful when dealing with samples containing limited cell numbers for traditional RNA sequencing, such as circulating tumour cells, early embryonic cells and some laboratory unculturable microorganisms. The Microwell‐Seq platform was constructed by the laboratory of Guoji Guo of Zhejiang University and applied to establish the world's first mammalian cell map.^29^ In general, it is essential for biologists to make a suitable choice according to the biological question and study design.

**TABLE 1:** Summary of scRNA‐seq methods. Several scRNA‐seq methods are listed and compared

Method	Drop‐seq/inDrop	Microwell‐seq	SmartSeq/SmartSeq2	MARS‐seq
mRNA reverse transcription	Template‐switching method	Template‐switching method	Template‐switching method	Second‐strand synthesis
cDNA amplification	PCR	PCR	PCR	In vitro transcription
Coverage	3′ or 5′ end of mRNA	3′ end of mRNA	Full‐length mRNA	3′ end of mRNA
Capture method	Droplet	Microwell	Plate	Plate
Cost	+ +	+	+ + + +	+ + +
Imaging capability	No	Yes	Yes	No
UMI	Yes	Yes	No	Yes
Reference	^21^	^27^	24, 25	^26^

The data analysis of scRNA‐seq is generally composed of three stages: primary analysis (base detection), secondary analysis (multiple separation, comparison and genetic identification) and tertiary analysis (data visualization and interpretation). Tertiary analysis should be given more attention mainly because of its complexity and importance for in‐depth insights into studies. There are many software programmes for data visualization and integration, such as Seurat, T‐Distributed Stochastic Neighbourhood Embedding (t‐SNE), Uniform Manifold Approximation and Projection (UMAP) and Monocle. Seurat, an R package for QC, analysis and exploration of scRNA‐seq data, enables users to find and identify sources of single‐cell heterogeneity and integrate them into different types of single‐cell data. T‐SNE is a computational technique for visualizing high‐throughput data by assigning a location to each data point in a two‐dimensional or three‐dimensional graph. It is often applied to visualize subgroups through datasets of scRNA‐seq. UMAP is a high‐dimensional analysis that is an alternative to t‐SNE, and its computation time is shorter. Monocle is a scRNA‐seq analysis software based on R language designed to determine cell development track design and is an ideal choice for experiments in which the beginning and end cell states are already known. In general, researchers can use these analysis methods flexibly as needed.^30^, ^31^, ^32^, ^33^

## 3 NEW INSIGHTS INTO IMMUNE REGULATION BY scRNA‐seq UNDER PHYSIOLOGICAL CONDITIONS

### 3.1 Profiling novel and rare immune cell types of normal organs

**FIGURE 1:**
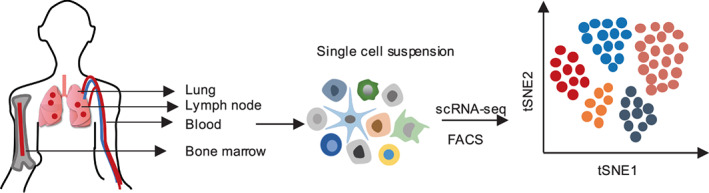
Profiling the immune cell atlas in normal organs. scRNA‐seq was applied to profile human T cells isolated from lungs, lymph nodes, bone marrow and blood.

The human body is composed of several organs, and these organs are all derived from a single cell, with immune‐related organs being no exception. However, a cell map of immune‐related organs has not been profiled. To obtain a novel understanding of normal organs, which is important for an understanding of the human body, scRNA‐seq for profiling immune cells can provide accurate insights into how the composition of immune cells affects organ development (Figure 1).

T cells belonging to the lymphocyte family play an important role in adaptive immunity in different tissue sites. However, it is still not clear how tissue sites influence their persistence and function. One representative example used scRNA‐seq to profile human T cells isolated from several tissues, including lungs, lymph nodes, bone marrow and blood. Meanwhile, their heterogeneity and functional responses to stimulation were revealed.^34^ In addition, scRNA‐seq was also adopted to identify the cellular distribution and heterogeneity of the lung in the process of development and dynamics. Additionally, distinct molecular markers have been uncovered, which indicates how whole‐tissue signalling interaction maps renewed our eyes on cellular networks at the single‐cell resolution in health and disease.^35^ From the above studies, the heterogeneity of T cells in different organs and novel cell types in mouse lungs were revealed, which provides a fresh view of immune cell heterogeneity‐related signalling interactions in different organs.

Although mice are widely used to map the immune cell composition of different organs, zebrafish is a kind of model organism that has been applied for many studies as well. A recent study used scRNA‐seq to profile lymphocytes of zebrafish during homeostasis and response to immune challenge. All of the cells were mapped from the gut of zebrafish, including wild‐type zebrafish and zebrafish lacking T and B cells. A type of cell expressing novel immune‐type receptors was discovered, and it probably functions as innate lymphoid cells (ILCs) with the recognition of environmental changes.^36^ In this study, novel molecular markers of zebrafish ILCs and a potential strategy for more deep exploration were revealed, which provides deeper insights into the immune regulation of zebrafish.

Generally, the results mentioned above have shown that scRNA‐seq can be applied to profile immune cells in different normal organs probably hidden in previous phenotypic studies, which will open the avenue for the appearance of novel ideas in future studies.

### 3.2 Reconstructing the trajectory of immune cell development

**FIGURE 2:**
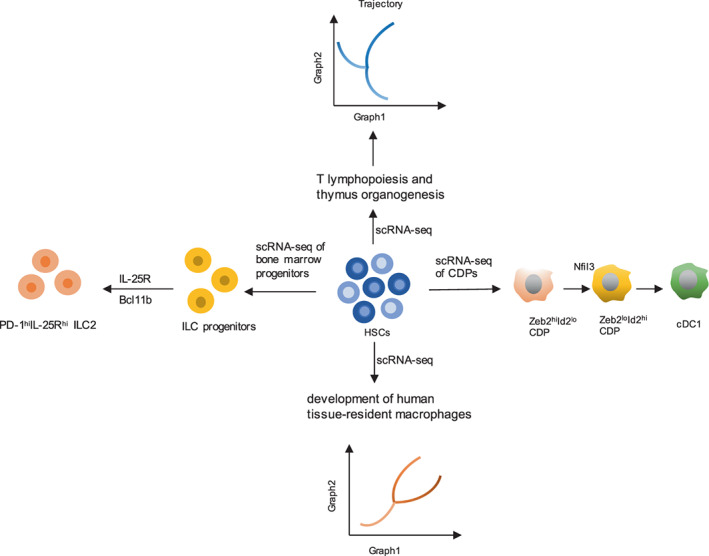
Reconstructing the trajectory of immune cell development in healthy states. scRNA‐seq of dendritic cell progenitors (CDPs) identified a cell type that expresses transcription factors influencing cDC1 development, and Nfil3 expression is required for Zeb2^hi^Id2^lo^ CDPs to transition to Zeb2^lo^Id2^hi^ CDPs, which represent the earliest committed cDC1 progenitors. In addition, the application of scRNA‐seq uncovered the development of ILC progenitors and revealed a cell type defined PD‐1^hi^IL‐25R^hi^ as an early checkpoint in ILC2 development, which was regulated by the zinc‐finger proteins Bcl11b and IL‐25R. In addition, the development of T lymphopoiesis and tissue resident macrophages was reconstructed by scRNA‐seq.

Most organisms start as a single cell that experiences several developmental stages to transitional and terminal cell types, many of which have yet to be defined. In recent years, with the wide usage of scRNA‐seq, some studies have used it to illuminate the developmental trajectory of particular immune cells and have discussed its influence on developmental biology (Figure 2).

Several studies have focused on the trajectory of immune cell development and the mechanisms that regulate the developmental process. For example, a recent study isolated cells from the thymus, aorta‐gonad‐mesonephros (AGM) region, liver and blood of human embryos and foetuses spanning embryonic and foetal stages to study early T lymphopoiesis and thymus organogenesis. It revealed distinct cell subsets of early thymic progenitors and the development of T lymphocytes, identifying molecular programmes that regulate cell development during the emergence, migration and thymus seeding of lymphoid progenitors. Another study profiled the thymus across the human lifetime and revealed the process of T‐cell development by scRNA‐seq, which will aid the establishment of in vitro organoid culture models.^37^, ^38^ In addition, Zhilei Bian and colleagues applied scRNA‐seq to profile CD45^+^ haematopoietic cells from human embryos at Carnegie stages 11 to 23 and characterized a population of CD45^+^CD34^+^CD44^+^ yolk sac‐derived myeloid‐biased progenitors (YSMPs) by single‐cell culture. They also constructed the developmental trajectories of tissue‐resident macrophages from either yolk sac‐derived primitive macrophages or YSMP‐derived embryonic liver monocytes, which provided novel insights into the development and molecular characteristics of human tissue‐resident macrophages.^39^

Developmental processes are driven by a series of transcriptional changes that allow for cell differentiation and commitment to a specific lineage and eventual cell type. Therefore, it is important to study the transcriptional changes specifically in the development of different immune cells. A recent study performed scRNA‐seq of the common dendritic cell progenitor (CDP). This study revealed novel cell subsets that influence type 1 conventional dendritic cell (cDC1) development through their transcription factors, such as Nfil3, Id2 and Zeb2. Further analysis of these factors indicated that Nfil3 plays an essential role in Zeb2^hi^Id2^lo^ CDPs transitioning to Zeb2^lo^Id2^hi^ CDPs.^40^ Yong Yu et al. focused on the cellular heterogeneity, developmental trajectory and signalling dependence of ILC progenitors. They performed scRNA‐seq of mouse bone marrow progenitors, delineating distinct ILC development stages and pathways. Furthermore, they revealed a committed ILC progenitor with high expression of programmed death 1 (PD‐1) and defined PD‐1^hi^IL‐25R^hi^ as an early checkpoint in ILC2 development, which was abolished by deficiency in the zinc‐finger protein Bcl11b but restored by IL‐25R overexpression.^41^

In general, these studies revealed the power of scRNA‐seq in reconstructing developmental processes and identifying several transcription factors regulating the transitions from one state to the next in immune cell development. Therefore, it can be widely used in reconstructing developmental trajectories of immune cells in any organ under both physiological and pathological conditions.

### 3.3 In‐depth understanding of immune cell type diversity and heterogeneity

Immune cells can be differentiated into several types according to their surface marker and location, but whether a certain cell type derived from the same progenitor cells possesses heterogeneity should be illuminated. Due to scRNA‐seq, many kinds of cells can not only be identified simultaneously but also have their heterogeneity and diversity revealed, and thus, it has been widely used in profiling immune cells. To identify different cell types according to their markers, there are many databases, such as CancerSEA,^42^ CellMarker,^43^ PanglaoDB,^44^ Cell BLAST,^45^ TISCH^46^ and Mouse Cell Atlas.^27^ They cover large numbers of cell types from humans and mice, which provides information on the first step in studies: cell type identification (Table 2).

**TABLE 2:** Common databases for identifying cell types and finding marker genes

Databases	Description	Websites	Reference
CancerSEA	The first dedicated database to fully explore the functional state of cancer cells at the single‐cell level	http://biocc.hrbmu.edu.cn/CancerSEA/; http://202.97.205.69/CancerSEA/	^42^
CellMarker	Large numbers of cell types included from human and mouse; supports the visualization and download of marker information	http://biocc.hrbmu.edu.cn/CellMarker/	^43^
PanglaoDB	Provides preliminary analysis results and a cell‐type marker compendium	https://panglaodb.se/	^44^
Cell BLAST	One‐stop solution for scRNA‐seq cell querying and annotation	https://cblast.gao‐lab.org	^45^
TISCH	A scRNA‐seq database focusing on tumour microenvironment.	http://tisch.comp‐genomics.org/	^46^
Mouse Cell Atlas	The first mouse cell map included from the same laboratory using microwell‐seq technology	http://bis.zju.edu.cn/MCA/gallery.html	^27^

**FIGURE 3:**
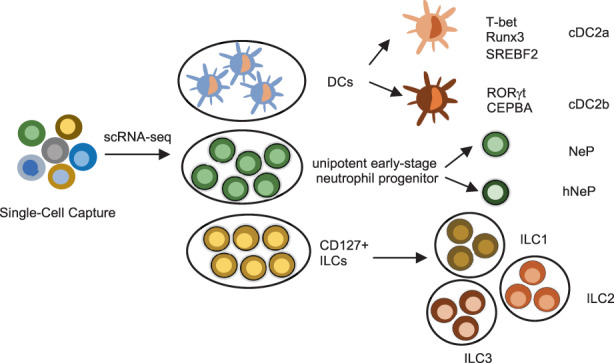
Character of the distinct immune cell subsets using scRNA‐seq. scRNA‐seq provides a novel perspective to reveal the heterogeneity of DCs, ILCs and neutrophils. Two principal cDC2 subsets, cDC2a and cDC2b, were identified by scRNA‐seq. Unipotent early‐stage neutrophil progenitors (NePs) and similar unipotent NePs (hNePs) in mouse and human bone marrow were discovered by scRNA‐seq. The heterogeneity of CD127^+^ innate lymphoid cells (ILCs), which include ILC1s, ILC2s and ILC3s, has not been characterized before.

Immunologists have gained insights into immune cell type diversity and heterogeneity to some extent. scRNA‐seq can be applied for studies of unknown immune cell subsets before and their molecular characteristics (Figure 3). For instance, a committed unipotent early‐stage neutrophil progenitor (NeP) and a similar unipotent NeP (hNeP) were discovered by scRNA‐seq in mouse and human bone marrow, respectively. In addition, NeP and hNeP were found to generate only neutrophils.^47^

Dendritic cells (DCs), sentinels of the immune system, reside in the blood and lymphoid tissues and are able to cross tissues of the whole body. They function by presenting antigens to T cells. There is a study combining scRNA‐seq and chromatin analyses to define two cDC2 subpopulations by particular transcriptional regulators called T‐bet and RORγt. These previously unreported cDC2 subsets were characterized by different metabolic and functional programmes.^48^

ILCs, a population of non‐T or non‐B lymphocytes, localize at mucosal interfaces and are able to regulate host defence and tissue homeostasis. The subpopulations of human ILCs (ILC1, ILC2, ILC3) were identified by scRNA‐seq of individual tonsil CD127^+^ ILCs, which provides a new understanding of ILC biology,^49^ with more exploration of the regulation of the immune system.

Thus, these studies provide a novel perspective into the heterogeneity of immune cells, including DCs, ILCs, neutrophils and monocytes. scRNA‐seq technologies are transforming our eyes to discover rare immune cell subtypes in their own niches.

## 4 PROBING IMMUNE CELL HETEROGENEITY AND REGULATION BY scRNA‐seq IN DISEASE STATES

### 4.1 Discovering a new regulatory mechanism of lymphocytes in different diseases

scRNA‐seq has been applied widely in cancer research during recent years mainly because of the high heterogeneity of tumours and the high resolution of scRNA‐seq. To better understand the correlation between immune regulation and the development of tumours, scRNA‐seq plays a significant role in studies, which may offer new therapeutic strategies and potential targets (Table 3). Because scRNA‐seq has been used for many tumour studies, we only discuss several examples below.

**TABLE 3:** Overview of studies applying scRNA‐seq to investigate immune regulation in pathological conditions

Species	Objects	Disease	Year	Reference
Human	T cells	Breast cancer	2018	^50^
Human	T cells	Hepatocellular carcinoma	2017	^51^
Human	T cells	Alzheimer's disease	2020	^52^
Mouse	T cells	Age‐related decline in brain function	2019	^53^
Human	T cells	Melanoma	2020	54, 55
Mouse	Th2 cells	Allergic airway disease	2019	^6^
Mouse	B cells	Dengue virus infection	2018, 2020	56, 57
Human	B cells	Follicular lymphoma	2019	^58^
Human	B cells	Breast cancer	2021	^59^
Human	B cells	Head and neck cancer	2020	^60^
Human	NK cells	Tuberculosis	2020	^61^
Mouse	NK cells	Solid tumours	2020	^62^
Human	NK cells	Oesophageal squamous cell carcinoma	2019	^63^
Mouse	Dendritic cells	*Propionibacterium acnes (P. acnes)* infection	2019	^64^
Mouse	Dendritic cells	Mouse cytomegalovirus infection	2020	^65^
Human	Dendritic cells	systemic lupus erythematosus	2020	^66^
Human and mouse	Dendritic cells	non‐small‐cell lung cancers	2020, 2019	67, 68
Mouse	Macrophages	Rheumatoid arthritis	2019	^69^
Human	Macrophages	Coronavirus Disease 2019	2020, 2021	14, 70
Mouse	Macrophages	Atherosclerosis	2018	^71^
Mouse	Macrophages	High‐fat diet‐induced obesity	2020	^28^
Human	Macrophages	Bladder cancer	2021	^72^
Mouse	Macrophages	Metastasis‐bearing lungs	2021	^73^
Human	Neutrophils	Coronavirus Disease 2019	2020	^75^
Mouse	Neutrophils	Allergic asthma	2019	^76^
Mouse	Neutrophils	Myocardial infarction	2020	^77^
Mouse, Human	Neutrophils	Lung cancer	2021	^78^
Mouse	Neutrophils	Liver toxicity in cancer immunotherapy	2021	^79^

#### 4.1.1 T cells

The process of adaptive immune responses requires precise spatial localization of B and T lymphocytes and antigen resent cells (APCs) in a dynamic manner. Specifically, scRNA‐seq in T cells demonstrates the potential power of these technologies to track antigen‐specific T‐cell clones, to study T‐cell subsets that function in different diseases and to probe molecular mechanisms and signalling pathways.

In a recent study, scRNA‐seq was performed to find T‐cell heterogeneity from human breast cancers (BCs), which revealed several distinct subsets of infiltrating T cells in which a cluster of tissue resident memory (TRM) T cells is likely related to improved survival rates of patients during the early stage of triple‐negative breast cancer (TNBC). Furthermore, it provides better prognostication than CD8 expression alone, which indicates that CD8^+^ TRM cells can positively affect BC immunotherapy and hopefully become targets of immune checkpoint inhibition.^50^ Zheng and colleagues performed deep scRNA‐seq of single T cells in hepatocellular carcinoma (HCC) patients. Specific subpopulations, such as exhausted CD8^+^ T cells and Tregs, are enriched and proliferate in HCC. Likewise, gene expression profiles for individual clusters were identified,^51^ which provides implications for the immune system and immune cell heterogeneity in cancers. Another study applied scRNA‐seq to map the blood and cerebrospinal fluid in Alzheimer's disease, which revealed a cluster of T cells patrolling the intrathecal space of brains.^52^ In addition, scRNA‐seq of neurogenic niches revealed a cell type of infiltrating T cells that can inhibit the proliferation of neural stem cells in mice, partly by secreting interferon‐gamma.^53^ Furthermore, scRNA‐seq of melanoma samples of patients discovered a subset of gamma delta T cells that are both overrepresented in the nonresponding tumours, which may provide a valuable tool for clinical decision making.^54^, ^55^

Some subtypes of T cells also play an important role in pathological states. For example, T helper 2 (Th2) cells can promote the pathogenic process of asthma, so, clear investigation of them mainly in cell heterogeneity is needed. A recent study conducted scRNA‐seq to identify T helper cells in the house dust mite (HDM) model of allergic airway disease. This result suggested that Th2 cells in the airways express many genes highly enriched in metabolism‐related signalling pathways, including glucose and lipid metabolism. Then, a series of experiments were performed and confirmed the results based on data analysis.^6^ These findings showed how Th2 cells and cytokines influence the pathogenesis of allergic asthma.

Collectively, these studies revealed the functional heterogeneity of T cells and their association with immunotherapy in cancers and clinical therapy in other types of disease, revealing unanticipated biological principles dictating poor outcomes in pathological processes.

#### 4.1.2 B cells

B cells are pluripotent stem cells derived from bone marrow. The mature B cells mainly reside in the superficial lymphoid nodule of the lymph node and in the red and white lymphoid nodule of the spleen. B cells can differentiate into plasma cells under antigen stimulation. In the last few years, many novel insights into B‐cell heterogeneity and its role in diseases have been uncovered by scRNA‐seq (Table 3). Here, we will discuss several examples that highlight how B cells function in disease conditions at single‐cell resolution.

A recent study used scRNA‐seq to profile large amounts of single PBMCs derived in the early course of disease infected with dengue virus (DENV). Naive B cells and monocytes have a high association with virus infection, which may be predictive cell populations of DENV infections.^56^ Ilka Hoof et al uncovered two B‐cell subsets of short‐lived IgE^+^ plasmablasts and IgG^+^ memory B cells that encode antibodies specifically for major grass pollen allergens.^57^ In addition, scRNA‐seq of 34,188 cells derived from six primary follicular lymphoma (FL) tumours revealed tumour‐specific features of malignant B cells compared with normal B cells from the same patient. Malignant B cells showed downregulated immunoglobulin light chain expression (either Ig Kappa or Ig Lambda) and an increase in the BCL2 gene but also a reduction in the FCER2, CD52 and MHC class II genes.^58^ Similarly, tumour‐infiltrated B cells in breast cancer have more mature and memory B‐cell features, higher proliferative ability and somatic hypermutations than those from peripheral blood.^59^ In patients with head and neck cancer, scRNA‐seq of the B cells revealed that they preferentially localized in the tumour stroma, indicating ongoing germinal centre reactions, which suggests that tumour‐infiltrating B cells could be harnessed for the development of therapeutic targets.^60^

Therefore, this approach can shed light on the role of B cells in diseases and promote the discovery of novel biomarkers of pathological processes.

#### 4.1.3 Natural killer cells

Natural killer (NK) cells are innate cytolytic and cytokine‐producing effector cells. They are activated upon signals originating from activating NK receptors, which are countered by inhibitory receptors specific for self‐MHC class I molecules. With the advent of scRNA‐seq, the cellular and molecular heterogeneity of NK cells have been studied more deeply and accurately in various diseases (Table 3).

In a recent study, scRNA‐seq analysis of peripheral blood mononuclear cells (PBMCs) from healthy controls (HCs), latent tuberculosis infection (LTBI) and active tuberculosis (TB) revealed a subset characterized by CD3^−^CD7^+^GZMB^+^ NK cells not reported before. Their proportion may become a potential diagnostic marker for TB, not LTBI. This kind of subset was also verified by flow cytometry in vitro.^61^ In addition, Jing Ni et al performed scRNA‐seq of mouse tumour‐infiltrating NK cells with or without conditional deletion of HIF‐1a, which showed that an enriched NK‐IL18‐IFNG signature in solid tumours correlated with increased patient survival rates. Thus, inhibition of HIF‐1α in tumour‐infiltrating NK cells could be applied for cancer therapy.^62^ Another study profiled a detailed immune cell atlas of oesophageal squamous cell carcinoma (ESCC) and found that NK cells are major proliferative cell components in the tumour microenvironment.^63^

### 4.2 Myeloid cells

#### 4.2.1 Dendritic cells

DCs are antigen presenting cells that play a vital role at the interface of innate and adaptive immune responses. DCs have been discussed in many studies that dissect DC subtypes based on their molecular characteristics, functions, tissue localization and developmental trajectory. However, the DC subtypes could be illustrated more specifically than before with the advancement of scRNA‐seq. Here, we present several pathological studies of DCs at the single‐cell level (Table 3).

DCs have been explored with scRNA‐seq in bacterial infections. For example, during *Propionibacterium acnes (P. acnes)* infection, dermal cDC1 s and cDC2s were examined by scRNA‐seq, which revealed that cDC1 s but not cDC2s regulate the degree of the immune response to *P. acnes* in the dermis of mice. This process is dependent on cDC1 s regulating neutrophil recruitment to the site under inflammation. Likewise, this finding was also uncovered in the process of *Staphylococcus aureus (S. aureus)*, Bacillus Calmette–Guerin (BCG) and *E. coli* infections.^64^ In another study, scRNA‐seq of animals infected with mouse cytomegalovirus revealed that IFN‐I production and T‐cell activation were performed by the same plasmacytoid dendritic cells (pDCs) but in different infected locations. IFN‐I production by pDCs was early identified by their downregulation of LIFR and activation by cell‐intrinsic TNF signalling.^65^

DCs have also been studied in other diseases. For instance, scRNA‐seq of pDCs in systemic lupus erythematosus (SLE) showed that Type III IFN production is triggered by RNA‐containing immune complexes (RNA‐ICs) in pDCs in a TLR‐MyD88‐dependent manner, which supported the contribution of both type I and type III IFNs in SLE.^66^ Moreover, a new cluster of DCs named mature DCs enriched in immunoregulatory molecules (mregDCs) because of their co‐expression of immunoregulatory genes (Cd274, Pdcd1lg2 and Cd200) and maturation genes (Cd40, Ccr7 and Il12b) was uncovered in human and mouse non‐small‐cell lung cancers by scRNA‐seq. They further demonstrated that upregulation of the programmed death ligand 1 protein in mregDCs is induced by the receptor tyrosine kinase AXL, which may be regulated by IL‐4 signalling.^67^ Another study applied scRNA‐seq of human and mouse lung cancers and revealed the heterogeneity of DCs (pDCs and cDC1‐2 subsets) across individuals and species.^68^

#### 4.2.2 Macrophages

Macrophages are composed of tissue‐resident and professional phagocytic cells that play a pivotal role not only in defence against pathogens but also in organ homeostasis. Recent studies have tried to deeply identify their tissue‐specific functions and various roles in immune‐related diseases at the single‐cell level, which may open an avenue for therapeutic strategies focused on macrophages (Table 3).

Macrophages are considered to contribute to rheumatoid arthritis, but how they affect inflammatory joint disease should be clearly investigated. A recent study used scRNA‐seq and found that membrane‐like structures contain a remarkable cluster of CX3CR1^+^ tissue‐resident macrophages, which formed an immunological barrier internally at the synovial lining,^69^ which revealed functional diversification among synovial macrophages not reported before.

In coronavirus disease 2019 (COVID‐19), immune cells of bronchoalveolar lavage fluid (BALF) from patients with varying severities of COVID‐19 were profiled by scRNA‐seq and showed that proinflammatory monocyte‐derived macrophages are abundant in patients with severe COVID‐19, while moderate cases are characterized by CD8^+^ T cells.^14^ Another study indicated that increased cell populations of macrophages expressing long pentraxin 3 (PTX3) were detected in patients with COVID‐19 by single‐cell sequencing and bioinformatics analysis.^70^

Atherosclerosis is a kind of chronic inflammatory disease involving the accumulation of macrophages in the vascular wall. Atherosclerotic arteries are characterized by many immune cells, including macrophage subsets that have not been clearly investigated due to restricted markers. To this end, a recent study using scRNA‐seq to profile CD45^+^ cells in healthy and atherosclerotic aortae found subsets of aortic macrophages and monocyte‐derived dendritic cells and identified previously unreported macrophage populations with novel markers.^71^ Therefore, it is significantly effective for researchers to find more markers and identify particular cell subpopulations.

Previous studies have shown that obesity increases the development of breast cancer, but the specific mechanisms affecting communication among cells in the tumour microenvironment remain clear. A recent study using scRNA‐seq to identify mammary immune cells indicated that high‐fat diet (HFD)‐induced obesity induces the generation and/or recruitment of M2 macrophages in mammary glands.^28^

For cancer immunotherapy, immune checkpoint blockade (ICB) has been a remarkable clinical advance for cancer; however, the majority of patients do not respond to ICB therapy. A recent study found a type of Tim‐4^+^ cavity‐resident macrophage by scRNA‐seq. It revealed that antitumour CD8^+^ T cells are susceptible to sequestration away from tumour targets and proliferation suppression by Tim‐4^+^ macrophages.^72^ In addition, extensive macrophage heterogeneity of lung metastases was revealed by scRNA‐seq. A novel subpopulation was identified with enriched genes involved in lipid metabolism and immunosuppression.^73^

Therefore, a comprehensive understanding of the different characteristics of macrophages driving disease pathogenesis is worth illustrating mainly in their heterogeneity at the single‐cell resolution, which may provide potential therapeutic targets for diseases.

#### 4.2.3 Neutrophils

Neutrophils are derived from haematopoietic stem cells in bone marrow and then enter the blood or tissue. They secrete proinflammatory cytokines, release neutrophil extracellular traps and produce reactive oxygen species in tissue injury conditions. The recent development of scRNA‐seq allows investigating the transcriptome of individual neutrophils in disease models, which supports cell subset identification and allows discovering more precise molecular mechanisms in diseases. Several examples are discussed as follows (Table 3).

Wilk and colleagues used scRNA‐seq to profile peripheral blood mononuclear cells (PBMCs) from patients infected with COVID‐19 and identified immune cell heterogeneity, including a developing neutrophil population that may be related to plasmablasts in patients with acute respiratory failure.^74^ In another study, eight neutrophil populations with distinct molecular signatures were identified by scRNA‐seq during bacterial infection. Bacterial infection reprogrammes the genetic architecture of neutrophil populations, alters the dynamic transition between each subpopulation and primes neutrophils for augmented functionality without affecting overall heterogeneity.^75^

For allergic asthma, scRNA‐seq of lung neutrophils in mice exposed to a proallergic dose of lipopolysaccharides (LPS^lo^) or a protective dose of LPS (LPS^hi^) before exposure to house dust mites (HDM) found that exposure to LPS^lo^ instructed recruited neutrophils to upregulate the expression of the chemokine receptor CXCR4 and to release neutrophil extracellular traps (NETs), which indicates that unrelated environmental risk factors can shape recruited lung neutrophils to promote the initiation of allergic asthma.^76^ In murine myocardial infarction, Ehsan Vafadarnejad et al employed scRNA‐seq to identify temporal neutrophil diversity. They provide a time‐resolved census of neutrophil diversity and gene expression dynamics and reveal a process of local tissue specification of neutrophils in the ischaemic heart characterized by the acquisition of a SiglecF^hi^ signature.^77^

For cancer immunotherapy, Veglia et al applied scRNA‐seq to identify the heterogeneity of polymorphonuclear neutrophils (PMNs) in cancer. They revealed the heterogeneity of neutrophils in multiple tissues, and the findings were conserved both in mice and humans, which provides potential targets for cancer immunotherapy.^78^ In addition, another study revealed that neutrophils upregulated an interferon‐responsive signature compartment in the tumour‐free liver following immunotherapy with a CD40 agonist, causing hepatotoxicity.^79^

Overall, these studies have helped to obtain a better understanding of infections and immune regulation mainly at the neutrophil level. These studies have revealed several regulators and molecular mechanisms that support the identification of treatment methods and potential therapeutic targets.

### 4.3 The cell–cell interaction among immune cells was revealed by scRNA‐seq

Tissue‐resident immune cells occupy a particular niche where surrounding cells produce the required chemokines to attract them and secrete the survival factors needed to remain alive. Similarly, immune cells arriving from the circulation system also interact closely with the vascular endothelial cells in the organ. In addition, several studies in recent years have shown that some tissue‐resident immune cells are able to interact closely and even directly by scRNA‐seq. For instance, Lavin et al combined scRNA‐seq and CyTOF (cytometry by time of flight) to generate an atlas of early lung adenocarcinoma parts. They found that T‐cell and NK‐cell compartments were altered significantly and that changes in tumour‐infiltrating myeloid cell (TIM) subsets may coordinate antitumour T‐cell immunity. Another study found that endothelial cells can downregulate immune cells. In addition, endothelial cells and related genes are coregulated with immune checkpoint transcripts, which are associated with T‐cell activation.^80^, ^81^ In addition, Elyada et al. employed scRNA‐seq and discovered a new kind of CAFs called ‘antigen‐presenting CAFs’ (apCAFs) expressing MHC class II and CD74, which activated CD4^+^ T cells in an antigen‐specific fashion, confirming their immune‐modulatory capacity in pancreatic ductal adenocarcinoma (PDAC).^82^

Other studies have focused on immune cell interactions and the molecular mechanisms regulating therapeutic strategies in pathological processes. In a recent study, scRNA‐seq was established and found that inhibited T‐cell activation correlated with pathological features clinically and key signalling pathways possibly modulating the development of tumour cell heterogeneity, which provides a potential resource for revealing intratumoral heterogeneity in PDAC, indicating an association between the intrinsic state of tumours and T‐cell activation.^83^ In addition, two scRNA‐seq technologies were used to profile CD45^+^ immune cells from five immune‐relevant sites in HCC patients. A novel type of LAMP3^+^ DC can migrate to lymph nodes (LNs) and exhibit the potential to regulate lymphocytes. Furthermore, myeloid and lymphoid cells in ascites were significantly associated with tumour origins.^84^ In addition, Turaj et al found that anti‐CD27/CD20 has a strong benefit in tumour immunotherapy. A detailed mechanism was revealed by scRNA‐seq, which illuminated that anti‐CD27 stimulated CD8^+^ T and NK cells to release myeloid chemo‐attractants and interferon gamma, which further induced myeloid infiltration and macrophage activation.^85^

## 5 CONCLUSION AND PERSPECTIVES

### 5.1 Advantages and the development trend of scRNA‐seq

In recent years, scRNA‐seq technology has been rapidly developed and widely applied in diverse areas of study, such as development, immunology, neurobiology, cancer and epigenetics. Of course, there are many advantages for scRNA‐seq, both technologically and biologically. In detail, scRNA‐seq has provided an unprecedented view of the repertoire of cell types present in a tissue or even in a whole organism and further insights into potential regulatory networks for biologists. In addition, scRNA‐seq represents a useful tool to investigate biological processes functionally and is able to uncover key information, including either healthy or disease states. It opens up a new way to more accurately understand human diseases in the process of diagnosis and treatment. Furthermore, the integration of scRNA‐seq and other single‐cell techniques, such as single‐cell spatial transcriptome sequencing and scATAC‐seq, has been applied in many recent studies and may be effective for the clear illustration of gene regulatory mechanisms at high resolution.^86^, ^87^, ^88^ However, the integration of single‐cell multimodel omics still faces many challenges, including complex workflows and high costs. In general, scRNA‐seq may be a valuable opportunity for studies of cell heterogeneity and deep exploration of more information, mainly due to its relatively low costs and high throughput.

With the advent and advancement of scRNA‐seq, it shows strong advantages in studying complex problems clinically and biologically. In the next few years, emerging technologies in the field of scRNA‐seq will probably be further performed in the research of immune‐related diseases, mainly in human samples. Single‐cell multiomics data, spatial transcriptome analysis techniques and the development of frozen sample analysis will provide more comprehensive information. The application of scRNA‐seq will help reveal the details of the occurrence and development of various biological processes, providing novel insights into pathogenesis, clinical therapy and drug development.

### 5.2 Challenges in scRNA‐seq

Despite these opportunities, scRNA‐seq technologies also face some challenges. First, the throughput should be increased, and improvements in sensitivity and accuracy should be noticed as well, mainly because of the mispairing and mutation that occur during reverse transcription and cDNA amplification. Second, computational methods must be improved. The main challenge is to effectively separate technical noise from the heterogeneity of gene expression levels driven by biological factors and analyse the integration of heterogeneous data from multiple sources. For example, when different data from scRNA‐seq and single‐cell spatial transcriptome sequencing should be integrated for analysis, the discordance of data features cannot be addressed well. Third, it does not carry the spatial information of a single cell, infer epigenetic landscapes and reflect the protein level. Therefore, it is essential to integrate scRNA‐seq with information from cytometry by CyTOF or fluorescence‐activated cell sorting (FACS) to identify whether this is true.^89^ Moreover, doublet, artifactual libraries generated from two cells often occur in automated scRNA‐seq experiments, resulting in suspicious biological conclusions. In addition, the high cost of scRNA‐seq is still a problem for biological studies of multiple samples. In general, there are still some challenges existing for scRNA‐seq, which need more efforts to improve its accuracy, doublets, bias and sequencing depth.

### 5.3 Conclusions

With the development of scRNA‐seq technologies, our view of many biological processes has been broadened. It plays a substantial role in many studies, not only immune regulation, which is introduced in this review but also in other areas of research. As scRNA‐seq becomes more easily available and is combined with other single‐cell technologies, genomics at the single‐cell level will become general and extremely powerful. However, there is a great need for further improvement in scRNA‐seq technologies. Future studies should also focus on how to validate the results of scRNA‐seq data by combining other single‐cell sequencing technologies or performing more experiments. Future technologies should focus on collecting combinations of genomic information from the same single cell in parallel. Above all, it is predictive that the application and development of scRNA‐seq will continue to improve extremely in future studies. It will become more sensitive and accurate, higher‐throughput, lower‐cost and more widely used in scientific research and clinical laboratories.

## AUTHOR CONTRIBUTIONS

**Xikun Zhou:** Validation (equal); writing – review and editing (equal). **Xueli Hu:** Writing – original draft (equal).

## FUNDING INFORMATION

This work is supported by the National Natural Science Foundation of China (No. 82172285 and 81922042), the National Clinical Research Center for Geriatrics, West China Hospital, Sichuan University (No. Z2018B02), the Innovative Spark Foundation of Sichuan University (No. 2018SCUH0032) and 1·3·5 project of excellent development of discipline of West China Hospital of Sichuan University (No. ZYYC21001).

## CONFLICT OF INTEREST

The authors declare that they have no conflicts of interest.

## Data Availability

Data sharing not applicable ‐ no new data generated.
